# Sustained Effects of Muscle Calpain System Genotypes on Tenderness Phenotypes of South African Beef Bulls during Ageing up to 20 Days

**DOI:** 10.3390/ani12060686

**Published:** 2022-03-09

**Authors:** Annie Basson, Phillip E. Strydom, Esté van Marle-Köster, Edward C. Webb, Lorinda Frylinck

**Affiliations:** 1 Animal Production, Agricultural Research Council, Private Bag X2, Irene 0062, South Africa; bassona@arc.agric.za; 2 Department of Animal Science, Faculty of Natural and Agricultural Sciences, University of Pretoria, Pretoria 0002, South Africa; este.vanmarle-koster@up.ac.za (E.v.M.-K.); edward.webb@up.ac.za (E.C.W.); 3 Department of Animal Sciences, University of Stellenbosch, Private Bag X1, Matieland, Stellenbosch 7602, South Africa; pestrydom@sun.ac.za

**Keywords:** SNP, calpain–calpastatin system genes, genomic association, tenderization, ageing

## Abstract

**Simple Summary:**

When searching for genetic markers for the selection of more tender beef, it is important to maintain minimal environmental variation from pre-slaughter, right through to the ageing process, to ensure the accuracy of the obtained phenotypes. This is because beef quality traits have a large environmental component that can greatly alter the characteristics of the meat, which would not reflect a true genetic effect. We propose that variable ageing times are especially important in determining whether markers are associated with tenderization or not. Our analyses included candidate genes for the protein degrading enzyme system for calpains, because they contribute the most to tenderization. We were able to validate these markers in South African beef cattle, where they could be useful for selection. The timing of the collection of tenderness data was critical, as only a few (6/134) genetic markers sustained their association with tenderization over ageing to 20 days. A larger tenderization effect earlier in ageing, as shown here for the *capn1_187* and *capn1_4751* markers, would decrease the length of ageing. This would not only increase profits, but also decrease the energy needed during the storage and refrigeration of aged beef, decreasing the carbon footprint of beef production.

**Abstract:**

The most important factor that determines beef tenderness is its proteolytic activity, and the balance between calpain-1 protease activity and calpastatin inhibition is especially important, while contributions can also arise from calpain-2 and, possibly, calpain-3. The meat ageing process itself affects these processes. To determine whether genotypes in the calpain–calpastatin system can enhance tenderness through a 20-day ageing period, South African purebred beef bulls (*n* = 166) were genotyped using the Illumina BovineHD SNP BeadChip through a gene-based association analysis targeting the *cast*, *capn3*, *capn2* and *capn1* genes. The Warner–Bratzler shear force (WBSF) and myofibril fragment length (MFL) of *Longissimus thoracis et lumborum* (LTL) steaks were evaluated between d 3 and d 20 of ageing, with protease enzyme activity in the first 20 h post-mortem. Although several of the 134 SNPs are associated with tenderness, only seven SNP in the *cast*, *capn2* and *capn1* genes sustained genetic associations, additive to the ageing-associated increases in tenderness for at least three of the four ageing periods. While most genomic associations were relatively stable over time, some genotypes within the SNP responded differently to ageing, resulting in altered genomic effects over time. The level of ageing at which genomic associations are performed is an important factor that determines whether SNPs affect tenderness phenotypes.

## 1. Introduction

Increased tenderness with beef ageing involves the weakening of the myofibrillar structure of muscle fibers through the degradation of myofibrillar (and other) proteins [1,2,3]. The proteolysis that occurs due to the muscle calpain system activity during ageing results in tenderization [4] and is mainly attributed to calpain-1 [2], with prominent modulating effects of calpastatin [5,6]. The contribution of calpain-2 protease becomes progressively more important as ageing progresses [7], while calpain-3 activity could also potentially contribute to the ultimate tenderness of beef [8], although this remains controversial. By extending the ageing period, tougher steaks that age at a slower rate can achieve greater levels of tenderness [9] and it is important in (for example) cattle with a larger *Bos indicus* genetic background [10], allowing for an improved quality before going to market. However, this results in logistical challenges of chilled storage and tracking large amounts of beef over extended time periods, whereas the ultimate goal is to accelerate these processes as much as possible [11] and shorten the ageing period [12].

The aim of selection to improve the tenderness of beef [13] goes hand in hand with the goal to accelerate the ageing process in order to reach the ultimate tenderness as rapidly as possible [11]. The efficacy of SNPs for selection for tenderness is different between populations [14], and there are genetic differences among beef breeds with regard to the calpain system and expression of tenderness. In South Africa, studies based on high-throughput genotyping have been limited and generally focus on genetic characterization and genomic relationships [15,16]. Studies on the genetic diversity and the application of genomic selection use moderate-density SNP arrays such as the 50K, [17,18,19,20], 80K [21,22,23] and 150K SNPs [16], but these could lack the sufficient SNP density for the characterization of indigenous beef breeds [23]. Although disease resistance SNPs [24] and potential beef quality SNPs have been identified [25], association studies using detailed tenderness phenotypes in the calpain–calpastatin system have only been performed with a very limited SNP pool [26].

Genome-wide association studies (GWAS) are useful for determining novel associations within phenotypes, while SNPs within targeted genes give a higher power of association of analysis for quantitative traits [27,28]. The use of candidate genes or gene-assisted selection is effective (given accurate phenotypic data), because even small gains in quantitative traits are economically valuable [29].

The duration of the ageing period may affect the estimates of heritability, with variations between breeds and ageing time, including interactions between these two variables [30]. This is likely related to the fact that there are differences in the rate of tenderization between different breeds and individual muscles [31], even when breeds are subjected to the same production conditions and/or carcasses to the same post-mortem processing procedures.

Accurate phenotypes are a prerequisite for linking genotypic data with traits related to beef quality [32], especially because many of the beef quality phenotypes have a moderate to low heritability [33,34]. Tenderness phenotypes under varying environmental conditions (perimortem and post-slaughter practices) are still poorly defined and large datasets with detailed phenotypes and appropriate SNPs in the causative genes are lacking. Studies in controlled environmental and slaughter conditions sometimes only include a small number of animals [35,36] and/or a small number of genotypes [35,37] due to the financial costs involved in raising and slaughtering animals. Other genomic association studies of beef quality have pooled data collected from different herds subjected to varying production systems [38,39,40], with or without electrical stimulation at varying ageing periods [38]. Large studies are often constrained by the cost to genotyping [41], so that the number of SNPs analyzed was sometimes limited to lower-density SNP chips, such as 7K–150K [42,43], inferred through imputation [40,44], or only a few individual SNPs from causal genes were used [35,37,38,39,45,46]. Data from *Bos indicus* using a limited *capn1* and *cast* SNP pool suggested that ageing time (<21 d) may alter genomic associations [45], while it has been suggested that ageing decays genetic associations with tenderness phenotypes over time [47,48]. Few studies have explored the effect of different post-slaughter practices such as the ageing period on the association between genotypes and tenderness phenotypes [39,45,49,50], and many studies used a limited number of single nucleotide polymorphisms (SNPs), while no data are available from South African beef cattle.

The augmentation of tenderness through post-mortem practices, such as electrical stimulation, tenderstretch or extended ageing, could eliminate differences in tenderness over time [9], altering genomic associations depending on the relationship between the sample collection and these factors [45], and making selection less effective. To determine whether extended ageing affects the genomic association between candidate SNPs and tenderness, we perform a regional, gene-based association study using the Illumina BovineHD (777K) BeadChip focusing on the genes of the calpain–calpastatin system over 20 days of ageing. This is the first study to explore the association between the BovineHD SNP of the *cast* (chromosome 7), *capn-3* (chromosome 10), *capn-2* (chromosome 16) and *capn-1* (chromosome 29) genes with tenderness (WBSF and MFL) up to 20 days of ageing in South African beef cattle. The objective of this study is to determine whether favorable genotypes in causative genes for tenderness could improve the phenotype over extended ageing up to 20 days and whether these associations are similar over ageing periods and between breeds.

## 2. Materials and Methods

### 2.1. Animals

Purebred bulls, approximately nine months old, were feedlot finished for approximately 120 days at the Animal Production Unit of the Agricultural Research Council (ARC-AP) unit in Irene, Pretoria, −25°54′ 28°12′, altitude ≥ 1475 m. Five breeds were included in the study (Table 1) as representative of breed types. Angus were representative of British *Bos taurus*, Charolais European *Bos taurus*, Brahman *Bos indicus*, Bonsmara as an indigenous *Sanga*-type composite breed and Nguni as the indigenous *Sanga*-type, *Bos taurus africanus*. Bulls were slaughtered to yield A2/A3-class carcasses [51,52], meaning zero permanent incisors, with lean to medium fatness, and were approximately 12 months old at slaughter. Standard management and slaughter practices were used to minimize the variation in tenderness induced by environmental factors, and the same feedlot, abattoir and similar carcass handling and ageing procedures were used for all bulls.

### 2.2. Slaughter and Sampling

Bulls were transported to the ARC-AP abattoir (3.9 km from the feedlot) and held overnight with access to water. Following captive bolt stunning and exsanguination, bulls were slaughtered, carcasses were halved and the right half of each carcass was electrically stimulated for 15 s (500 V peak, 5 ms pulses at 15 pulses/s) and chilled directly (ES). The left half of each carcass served as a control and chilling was delayed for 6 h post-mortem (NS) to allow for accelerated conditioning at 10 °C before chilling.

Association analyses were originally performed on the left half and the right half of the carcasses separately, as well as the pooled data. Of all the association analyses performed (NS, ES, NS + ES) for each SNP and phenotype, only 0.3% exhibited genotype × treatment interactions (results not shown) and did not include any of the candidate SNPs identified here. In order to simplify data representation tables, only data from the NS + ES analysis were represented here and any notable differences between association analyses described in the text.

Meat or tail hair samples were collected at slaughter. Meat samples were stored at −20 °C until extraction of DNA from a core sample, while tail hair was stored at room temperature until extraction of DNA from hair bulbs. At approximately 20 h post-mortem, the *Longissimus lumborum et thoracis* (LTL; loin) muscle was excised (boned out). Several 30 mm steaks were collected from predetermined regions of the LTL for each of the laboratory analyses for each ageing period, vacuum-sealed (70 microns) and aged for three, nine, fourteen and twenty days post-mortem at 2 ± 1 °C (d 3, d 9, d 14, d 20). The myofibril fragment length (MFL) was determined following ageing, while steaks for the determination of Warner–Bratzler shear force (WBSF) were frozen at −20°C until analyzed. Small sub-samples were collected from the lumbar portions of the LTL at 1 h and 20 h post-mortem, snap-frozen to -196 °C in liquid nitrogen and immediately stored at −80 °C until analyses of muscle calpains and calpastatin activities.

### 2.3. Tenderness

For WBSF measurements [53], steaks were thawed overnight, oven-grilled at 200 °C to a core temperature of 70 °C and cooled to 18°C for core sample collection. A handheld cork borer (12.7 mm diameter) was used to collect six round, uniform cores from each steak, parallel to muscle fiber direction. Core samples were sheared through the center, perpendicular to the long axis of the muscle fibers with a Universal Instron apparatus (Instron Ltd., Buckinghamshire, England) fitted with a Warner–Bratzler V-notched shear blade (60° angle) [54]. The crosshead speed of the Instron apparatus was set to 200 mm/min and the peak force (kg) for each core was determined.

The MFL was determined following extraction according to Culler et al. (1978), as modified by Heinze and Brüggemann (1994) [1,55]. The mean of 100 fragments per steak (or ageing period) was determined using Video Image Analysis (VIA) with an Olympus System microscope, model BX40 (Tokyo, Japan) at a 400× magnification equipped with CC12 video camera.

### 2.4. Muscle Calpain Protease System

Calpain-1, calpain-2 and calpastatin were extracted from muscle tissue samples and separated by 2-step gradient ion exchange chromatography [56]. Using azo-casein substrate [57], a unit of calpain protease activity was defined as a 1.0/h increase in absorbance at 366 nm (*A*_336_) at 25 °C, while protease inhibition by calpastatin was defined as the amount required to inhibit one unit of calpain-2 activity. Enzyme activities and inhibition were expressed as units per grams of meat, while relative units of inhibition by calpastatin per units of calpain-1, or per the combined protease activities of calpain-1 and calpain-2, were also calculated.

### 2.5. DNA Extraction and Genotyping

DNA was collected from muscle or hair samples and extracted using the NucleoSpin^®^ Tissue kit (MACHEREY-NAGEL GmbH & Co. KG, Düren, Germany) with silica membrane columns, including modifications to the basic protocol to enhance DNA yield, as per the user manual. Samples were allowed to stabilize overnight at 4 °C and DNA quality and concentration were determined at the Agricultural Research Council, Animal Genetics (Irene, South Africa) using UV spectrometry (NanoDrop2000/2000c, Thermo Fisher Scientific, Waltham, MA, USA), fluorometry (Qubit^®^ 3.0, Thermo Fisher Scientific, USA) and ethidium bromide agarose gel electrophoresis.

All the animals were individually genotyped using the BovineHD SNP BeadChip (Illumina, San Diego, CA, USA) using the HiScanSQ platform and Genome Studio software (Illumina, USA) at the Agricultural Research Council, Biotechnology Platform (Pretoria, South Africa). The National Centre for Biotechnology Information (NCBI) Genome Data Viewer [58], using bovine genome assembly UMD3.1.1, was used to determine the extent of the four muscle calpain system gene transcripts (gene ± 8% length). The number of variants that fell within the range for each gene (before quality control) were 12 for the *capn1* gene (chromosome 29, 44,223 bp), 27 for *capn2* (chromosome 16, 70,008 bp), 18 for *capn3* (chromosome 10, 68,507 bp) and 77 for *cast* (163,722 bp). For each SNP, the name was abbreviated as the gene plus the last three digits of the Illumina code (*gene_123*), except for the SNP *capn1-4751*, which was abbreviated from the Illumina CANP1_2 to *capn1_4751*. The allele that favored tenderness was determined by comparing phenotypic means of the three genotypes, first for the genotype with the lowest WBSF and then MFL (if necessary), where these alleles were indicated with an asterisk and designated the tender allele (*A/*C/*G/*T) that coded for the tender form of the protein. This simplified designation of the alleles was possible because no dominance effects were found in the candidate SNP effects.

Quality control was performed on the SNP using PLINK version 1.9 software [59] to exclude the SNPs with a genotyping (call) rate of <0.95, deviation from Hardy–Weinberg distribution (*P*_HWdev_ ≤ 1 × 10^−4^) and minor allelic frequency <5%. Five SNPs were disqualified during quality control, two from the *capn3* gene, two from the *capn2* gene and one from the *cast* gene.

### 2.6. Statistical Analyses

A mixed linear model association (mlma) analysis [60] was conducted to determine the association of genotypes with the tenderness phenotypes using GCTA 1.92.2 software [61] (University of Queensland, Queensland, Australia). The simplified mixed model (α = 0.05) used the genotype of the different SNPs (*bx*) as a fixed effect in a linear model, with a variance-standardized genomic relationship matrix (*g*) as a random accumulated effect of all SNPs and the residual (*e*) or random effect of error. To determine whether these responses were similar between breeds, associations were determined within breeds, with all breeds as covariates or with each individual breed added to the simplified model as covariate fixed effects (*covar_Br_*):

*y* = *a* + *bx* + [*covar_Br_*] + *g* + *e* (1)

We also performed an adjusted association analysis [62] on the simplified model. The false discovery rate as a result of multiple testing using GCTA was 24% for 134 SNPs in the traits associated with tenderization. When the adjustment in the *p*-value affected the results, these false positive associations were clearly indicated in tables and described in the results (*p*-value).

To determine the least squares means (LSM) of genotypes or genotypes within breeds, Statistical Analysis System (SAS Institute, Cary, NC, USA) software was used [63] to determine the least significant difference (α = 0.05). A generalized linear model procedure was conducted, including the effects of genotype (*Ge*) of the SNP, electrical stimulation treatment (*Tmt*), breed (*Br*), first-order interactions with genotype as well as residual (*e*):

*y* = *μ* + *Ge* + *Tmt* + *Br* + *GeTmt* + *GeBr* + *e* (2)

Because individual SNPs in a gene are not independent (genotypes of different closely associated SNPs are linked in inheritance or pleiotropy), a linkage disequilibrium (LD) analysis was performed using Haploview 4.2 software (Broad Institute, USA) [64] default analysis [65]. The LD indicates a link between genotypes (alleles at different loci) that was greater than that attributed to chance, i.e., LD [66,67]. This implies that, through this association, selection for one SNP with a high level of LD with another SNP would indirectly result in selection for the other SNP, as they are not genetically independent.

SNPs with Lewontin’s D-prime (D′) that was greater than the threshold of 0.95 were likely to also be affected when a candidate SNP was included in a selection program. All SNPs that were identified as dependent on a candidate SNP that had additive effects through extended ageing were also subjected to the gene-based association analyses to ensure the direction of change in tenderness phenotypes was also positive and that no (readily apparent) inadvertent negative effects were linked to the SNP that sustained tenderness over extended ageing.

## 3. Results

### 3.1. Tenderness

**Figure 1:**
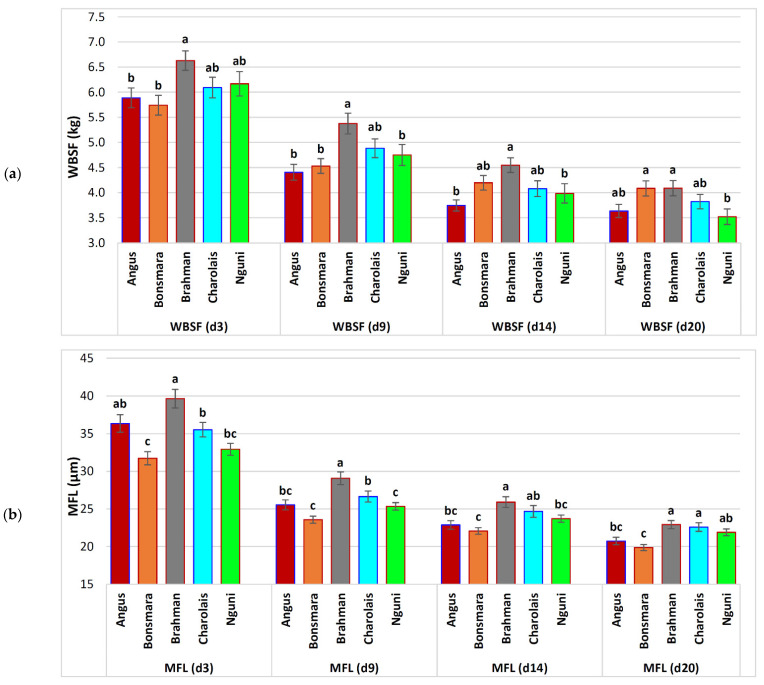
Changes in tenderness in five South African beef breeds over an ageing period of 20 days. Least squares means (LSM) that were statistically significantly different within ageing periods (*p* ≤ 0.05) are indicated with different ascending letter superscripts (a, b, c). Error bars represent the standard errors of these LSM: (**a**) WBSF—Warner–Bratzler shear force; (**b**) MFL—myofibril fragment length.

On d 3 post-mortem, Brahman cattle had the toughest beef based on the highest WBSF values, while Bonsmara was the most tender, but was not different from Angus (Table 1; Figure 1a). The WBSF of Charolais and Nguni was intermediate in toughness. All breeds experienced a significant increase in tenderness between d 3 and d 9 of between 19% in Brahman and 25% in Angus. This was followed by another 15–16% decrease in shear force values to d 14, except Bonsmara where the decrease in the WBSF (i.e., improvement in tenderness) was only 7%. Between d 14 and d 20, there was little or no change in the WBSF in Angus and Bonsmara (3% lower), a moderate tenderization in Charolais (6% lower) and continued tenderization in Brahman (10% lower) and Nguni (12% lower WBSF). This meant that Nguni had the most tender beef on d 20 and the WBSF was 13.9% lower than Bonsmara, while Brahman had the highest WBSF scores at this stage of ageing (and Angus and Charolais had intermediate values).

The MFL on d 3 was the shortest in Bonsmara and longest in Brahman (Figure 1b). The MFL in Nguni, Angus and Charolais was similar in the myofibril length, where Nguni did not differ from Bonsmara (intermediate–short) and Angus did not differ from Brahman (intermediate–long). All breeds experienced a large decrease in the MFL between d 3 and d 9 post-mortem, which related to 23% in Nguni and 30% in Angus. The reduction in the MFL to d 14 was 10% in Angus and 11% in Brahman, where the decrease in the length of other breeds was 6–7%. On d 20, the MFL was still decreasing from d 14 values by between 8% in Nguni and 10% in Brahman. Although Brahman had the greatest absolute decrease in myofibril lengths (16.72 µm shorter), the long initial MFL was not overcome by ageing and it remained the highest of all breeds (with Charolais) on d 20 of ageing. In Bonsmara, the MFL remained the shortest throughout the entire ageing period (−12.7% of Brahman and Charolais), while Nguni had an intermediate–high MFL (3.8% shorter) and Angus an intermediate–low MFL (8.9% shorter MFL) on d 20.

### 3.2. SNP

Ten candidate SNPs were identified in this study that could affect tenderness over extended ageing periods for at least three out of the four ageing periods measured (from a pool of 134 SNPs in the four genes). Of these, one SNP was eliminated in quality control measures due to a very low minor allelic frequency, which could result in false positives. Another two SNPs, one in the *cast* gene and one in the *capn2* gene, were statistically significant but biologically negligible, as the differences in the LSM between genotypes were very small.

#### 3.2.1. *cast_736*

The *cast_736* SNP (rs137217365) was located 28.81 kb from the start of the *cast* gene. The SNP was independent, i.e., it did not exhibit LD (D′ > 0.95) with any of the other SNPs of the *cast* gene. It seemed, therefore, that there should not be any unintended effects of non-random inheritance from nearby loci. The genotype that favored tenderness, or the tender genotype *GG, was relatively rare, and occurred in only 19 of the 166 bulls (11%). However, data from bulls with the heterozygous genotype showed intermediate tenderizing effects, confirming the effects of the *G allele.

The *cast_736* genotype (Table 2) was associated with biologically small changes in the WBSF in the early ageing periods on d 3 and d 9 post-mortem (≤5.1%), but resulted in a larger decrease in shear force on d 14, while the tenderizing effect on d 20 was not statistically significant (*p* ≤ 0.1343). The 0.43 kg decrease in shear force on d 14 in *GG bulls was additive to a tenderizing effect of ageing that resulted in an almost 2 kg decrease in shear force values in all three genotypes. This meant that the tender genotype was still able to induce a 10.3% increase in tenderization in the face of the existing tenderization of ageing at 14 days of 36.0% compared to d 3. The addition of breeds to the simplified model as covariates, each breed as a covariate or within-breed analyses suggested that the effects of this genotype on the WBSF were not breed dependent. However, false discovery rate (FDR) analyses showed that these biologically small effects could be overestimated because of multiple testing, rather than a true association. The effects of the *cast_736* SNP on d 3 and d 9 only tended to be significant in the FDR analysis, while the association on d 14 was not significant (*p* ≤ 0.2340).

The *cast_736* SNP was associated with an MFL over the ageing period between d 9 and d 20, with little advantage to adding breed to the model. However, the phenotypic response to genotypes was greatly altered by the time of the ageing period. The MFL on d 9 tended to reach significance, but the response in the *GG genotype was an increase in the myofibril length of 1.70 µm, which would not favor tenderization. At this ageing period, the only significant breed-dependent effect was identified in Charolais, which was the only breed to exhibit a decrease in the MFL of 6.9% or 1.94 µm (within-breed *p* ≤ 0.0404). On d 14 post-mortem, the genotypes failed to result in biologically important changes in the MFL compared to TT (+0.9%), but was 4.2% shorter than GT. The association of the MFL with genotypes on d 20 resulted in a relatively small decrease in myofibril length (1.01 µm shorter) that occurred in addition to the large tenderizing effects induced by ageing. During the period where the *cast_736* SNP affected the MFL (d 9–d 20), ageing resulted in a 4.53 µm decrease in myofibril lengths in TT bulls and 4.10 µm in GT, while, at the same time, *GG bulls experienced a 7.24 µm decrease in MFL.

The lack of a consistent response in the MFL and the small changes in the WBSF could be explained by the differences between genotypes in the muscle protease system. The genotype did not affect calpain protease activities, while small and inconsistent associations were noted for calpastatin inhibition at 1 h post-mortem only (Table 2). The LSM of absolute calpastatin inhibition at one hour was not different between genotypes and was identified as a false positive association when *p*-values were adjusted for multiple testing, but tended to associate with inhibition in the mixed linear model. The relative inhibition of calpain-1 by calpastatin at 1 h was decreased by 5.9% in *GG bulls, while the decrease per units of calpain-1 and calpain-2 activity was biologically negligible. The effects on relative calpastatin inhibition remained significant in the adjusted association analysis.

#### 3.2.2. *cast_763*

The *cast_763* SNP (rs135465452) was located 98.91 kb from the start of the gene and was in strong LD (D′ > 0.95) with several SNPs associated with tenderness phenotypes (results not shown). When determining whether *cast_763* would be a suitable candidate for selection for tenderness that was sustained in the face of ageing, it was important to note whether the closely-linked SNPs also induced positive tenderizing effects, as selection for the candidate SNP would indirectly result in selection for SNPs in high LD. These SNPs included *cast_741* (−50.0 kb from cast_763), *cast_770* (+17.2 kb from cast_763), *cast_771* (17.9 kb), *cast_772* (18.9 kb) and *cast_779* (36.9 kb). Although none of these SNPs were identified as candidate SNPs for the improvement of tenderness over extended ageing periods, they were found to have associations with some tenderness phenotypes (unpublished results). The corresponding alleles for these SNPs were also favorable for tenderness and the general effects of these SNPs were mild reductions in the WBSF between d 3 and d 14 (of between 6.7 and 9.0%), with generally small improvements in the MFL on d 14 (5.0–6.5%).

The *cast_763* SNP did not associate with the WBSF at any of the ageing periods, but was associated with the MFL throughout the entire ageing period between d 3 and d 20 of ageing in the simplified model (Table 3). Although the responses in the MFL at these ageing periods were biologically small (4.4–5.5%), the differences between the LSM for the tender *CC genotype reached significance compared to CT. The tenderizing effect of the genotype was additive to an already-tenderizing effect of ageing on the MFL between d 3 and d 20 of 39% for both genotypes. There was no advantage in the addition of breed(s) to the simplified model.

The small differences in the myofibril length could be ascribed to small changes in protease enzyme inhibition that tended to occur. These differences were not consistently present and could be false positive associations (Table 3). The absolute calpastatin inhibition at 1 h post-mortem associated with the genotype was 5.5% lower, while relative calpastatin per combined calpain-1 and calpain-2 proteolysis at 20 h was 5.6% lower in the *CC genotype compared to CT. Although a tendency towards a decrease in calpain-1 at 1 h was observed, the difference was biologically negligible (a 1.4% shorter MFL) and could be considered unaffected by the genotype.

#### 3.2.3. *capn2_780*

The *capn2_780* SNP (rs135646764) was located 55.07 kb from the start of the gene. The SNP was in LD (D′ > 0.95) with other SNPs that affected tenderness in the gene-based association study, *capn2_760* (−46.93 kb), *capn2_763* (−35.72 kb), *capn2_766* (−26.53 kb) and *capn2_772* (−21.40 kb). Selection for the tender allele of the *capn2_780* SNP would include indirect selection for these SNPs, all of which were linked to the corresponding tender alleles. These SNPs were found to have no association with the WBSF or MFL, but did affect changes in enzyme function, which was a general increase in calpastatin and calpains, where calpain proteolysis increased to a greater extent than calpastatin inhibition. This resulted in a decrease in the relative calpastatin inhibition per units of calpain proteolysis, which would favor tenderization (results not shown). The tough GG genotype was relatively rare (*n* = 15), where 10 of these bulls were Brahman, while also not being sufficient for determining the LSM. The G allele was rare in Angus, with only three heterozygotes and no GG bulls. Because few “tough homozygotes” were available for comparison, genotype differences were compared between *AA (*n* = 112) and AG (*n* = 39), with the exception of the Angus breed, where the association could not be tested (minor allelic frequency = 0.06).

**Figure 2:**
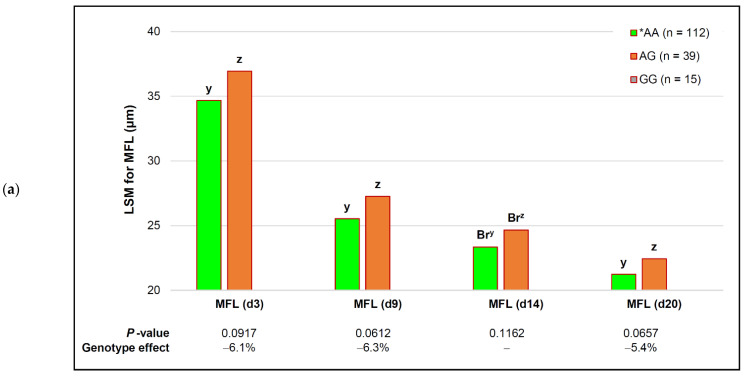

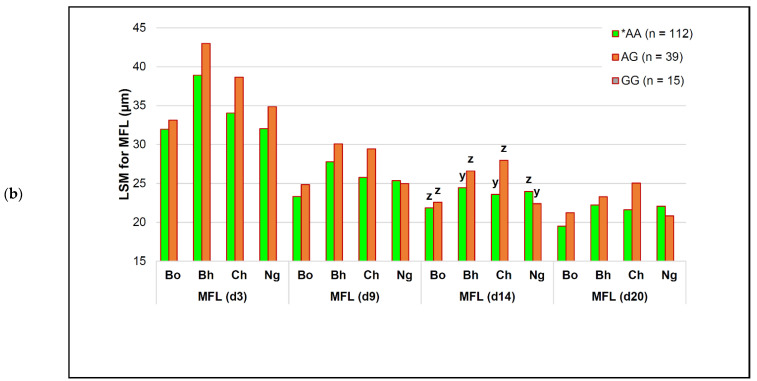
Additive effects of the *capn2_780* genotype and ageing on myofibril fragment length (MFL). *AA—the genotype that favored tenderness. Within each ageing period, different descending letter superscripts (z, y) tended to be significantly different (*p* ≤ 0.10): (**a**) genotype and ageing effects data pooled for all breeds; Br—different only when breeds were added to the simplified model; (**b**) genotype and ageing effects on MFL for individual South African beef breeds. Bo—Bonsmara; Bh—Brahman; Ch—Charolais; Ng—Nguni.

There were no associations between genotypes and the WBSF at any of the ageing periods (Table 4), while the *capn2_780* genotype tended to affect the MFL throughout the ageing period (Figure 2a). The tenderizing effect of the SNP on the MFL on d 14 was only apparent when breed(s) were added to the simplified model as covariate(s). The tender, or *AA genotype resulted in a >6% reduction in the MFL compared to AG on d 3 and d 9, which was sustained as a small 5.4% decrease on d 20. Although these tenderizing effects were biologically small, they were additive to a 39% decrease in the MFL already induced by ageing in both genotypes (Figure 2a).

The covariate effect of breed(s) was only present on d 14 post-mortem (Figure 2b), where the Bonsmara MFL remained unchanged, while tenderizing effects of the genotype were moderate in Brahman (8.1%) and large in Charolais (15.6%). The *AA genotype in Nguni bulls resulted in a 6.9% increase in myofibril lengths, or 1.54 µm, which was not a tenderizing response.

The fact that the consistent decrease in the MFL over the ageing period was not accompanied by a concomitant decrease in shear force values at any of the ageing periods was most likely due to a lack of changes in the proteolytic enzymes in the different genotypes (Table 4). The adjusted analyses (FDR) did not change the results of the multiple associations.

#### 3.2.4. *capn1_184*

The *capn1_184* SNP (rs17871986) was located 4.51 kb from the start of the gene and exhibited a high level of LD (D′ > 0.95) with three other SNPs associated with tenderness within close proximity of the SNP. The selection for the tender *G allele of *capn1_184* favored selection for the “tender alleles” for these SNPs that were closely linked to it (unpublished results). The *capn1_183* (−0.76 kb), *capn1_316* SNP (+1.10 kb) and *capn1_185* (+2.96 kb) SNPs were all associated with increased tenderization, with similar or smaller means differences than those discussed below. There was a shortage of tough homozygous bulls (AA) in the *Bos taurus* and Sanga types and a lack of tender homozygotes (*GG) in Brahman. However, the addition of breed(s) as covariate(s) did not improve the simplified mixed model in any of the phenotypes tested. A greater pool of animals would be required to fully elucidate the breed differences, if any exist.

**Figure 3:**
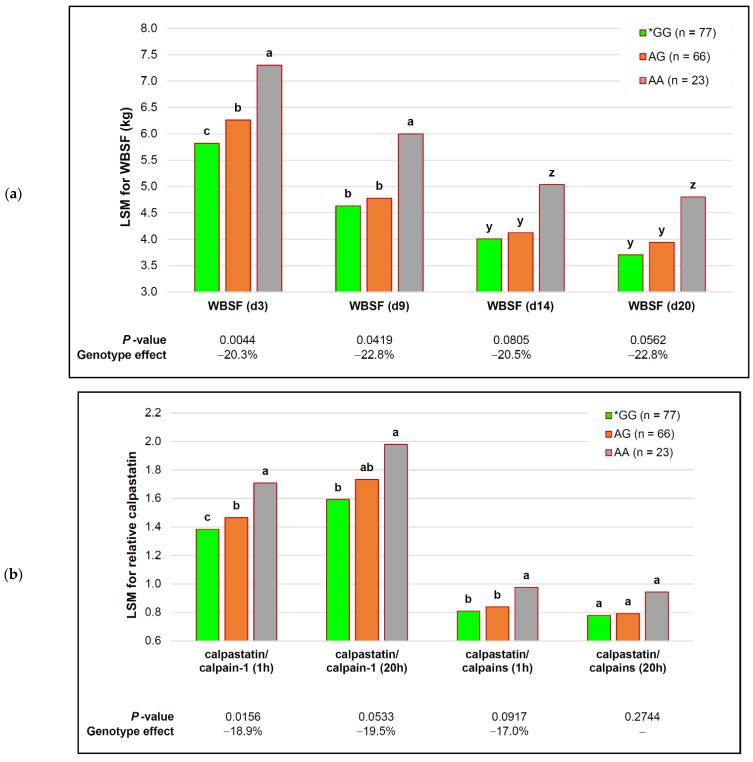
Additive effects of the *capn1_184* genotype and ageing on tenderness in representative purebred South African beef bulls. *GG—the genotype that favored tenderness. Within each ageing period, different ascending letter superscripts (a, b, c) were significantly different (*p* ≤ 0.05), and those that tended to be different (*p* ≤ 0.10) were indicated with different descending letter superscripts (z, y): (**a**) Warner–Bratzler shear force (WBSF); the effect on d 20 was not significant after FDR adjustment (adjusted *p* ≤ 0.1250); (**b**) relative calpastatin inhibition of protease(s).

The *capn1_184* SNP exhibited a strong, stable association with the WBSF over the entire ageing period (Figure 3a) and during intermediate ageing (d 9–d 14) for the MFL (Table 5), resulting in large phenotypic responses ≥19%). The differences between means observed for the different genotypes sustained levels greater than 1 kg shear force throughout the different ageing periods, where a 20.3% decrease in the WBSF of d 3 was maintained throughout, and up to 22.8% on d 20. The effect on the WBSF on d 20 was likely ascribed to multiple testing (adjusted *p* ≤ 0.1250) and this effect would need to be confirmed in a larger dataset. These genotype associations were additive to decreases in the WBSF of between 34.2 and 37.1% in all three genotypes over the ageing period (Figure 3a). Similar results were observed for the MFL at d 9 and d 14 of ageing, where myofibril lengths in *GG bulls were 7.31 µm and 8.04 µm shorter than AA bulls, respectively.

Large, consistent decreases in the WBSF and large, but less consistent, shorter MFLs were likely the result of the associations of the *capn1_184* SNP with the inhibition of calpastatin relative to protease activities (Figure 3b). The calpastatin per calpain-1 at 1 h and 20 h decreased by ≥19%, while calpastatin per combined calpain-1 and calpain-2 activities at 1 h was 17% lower. The LSM for heterozygous bulls was intermediate for calpastatin per unit of calpain-1 proteolysis, but not different from the tender genotype for inhibition relative to the combined proteolysis of calpain-1 and calpain-2. This would allow for an increased degradation of muscle proteins, greatly favoring the tenderization of beef.

#### 3.2.5. *capn1_187*

The *capn1_187* SNP (rs135658374) was located 17.59 kb from the start of the gene and was in strong LD (D′ > 0.95) with the *capn1_4751* SNP (+6.57 kb) discussed below. The distribution of alleles for Brahman was skewed toward the tough allele, while other breeds generally experienced a shortage of CC genotypes. The discussion, therefore, focused on the differences between the tender homozygous bulls (*TT) and heterozygotes (CT) for breed dependence, although there was little or no advantage to adding breeds to the simplified model as covariates.

**Figure 4:**
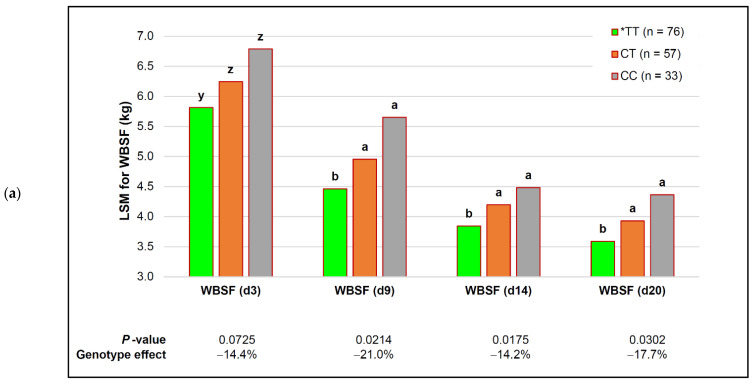

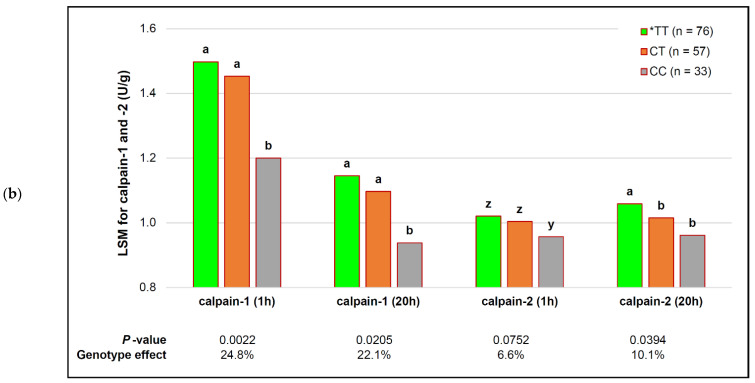
Additive effects of the *capn1_187* genotype and ageing on tenderness in representative purebred South African beef bulls. *TT—the genotype that favored tenderness. Within each ageing period, different ascending letter superscripts (a, b) were significantly different *p* ≤ 0.05), and those that tended to be significantly different *p* ≤ 0.10) were indicated with different descending letter superscripts (z, y): (**a**) Warner–Bratzler shear force (WBSF); the effect on d 20 only tended to be significant after FDR adjustment (adjusted *p* ≤ 0.0558); (**b**) calpain protease activities.

The SNP was associated with the WBSF over the entire ageing period. The least squares means differences between homozygous bulls were very close to 1 kg on d 3 and d 9 post-mortem (0.43–0.49 kg for heterozygous bulls), and the large gain in the tenderness of early ageing was decreased somewhat by d 20, where the shear force improved by 0.77 kg in *TT and 0.34 kg in CT. These genotype effects on shear force occurred additive to the 37% reduction in the WBSF that occurred in all genotypes by d 20 post-mortem (Figure 4a). Adjusting for multiple testing during association, the adjusted *p*-value for the WBSF on d 20 decreased from a significant *p* ≤ 0.0302 to a tendency *p* ≤ 0.0558.

The association of the *capn1_187* genotype with the MFL decayed over the time of ageing, with the most prominent effect on d 3, where an 11.78 µm decrease in length was observed in *TT bulls (26.6% shorter). As the ageing period progressed, the improvement in the MFL gradually decreased and was 5.42 µm (18.2%) on d 9 and non-significant by d 14.

There was a tendency toward a breed-dependent association of the *capn1_187* SNP and absolute calpastatin at 20 h (Table 6). A large increase in the absolute inhibition (toughening) was observed in Angus, Brahman and Charolais (+13–15%), with no effect in Bonsmara and a moderate tenderizing response (11% lower calpastatin) in Nguni. Although this apparent toughening effect was concerning, the adjustment of the *p*-values for multiple testing identified it as a potential false positive association (adjusted *p* ≤ 0.1607). The means differences for relative calpastatin activity per calpain(s) were, generally, unaffected, while the relative calpastatin inhibition per calpain-1 at 1 h was tenderizing (15.5% lower).

The large consistent responses in tenderization (especially the WBSF) were, therefore, not in response to the decreased calpastatin inhibition of protease activities, but rather the large and consistent responses of the proteases themselves to genotypes (Figure 4b). Increased protease activity in genotypes that contained the *T allele were large for muscle calpain-1 activities (17—25%), with moderate tenderizing effects in calpain-2 (≤10%). These increased rates of proteolysis would favor myofibrillar fragmentation, decreasing the length of the fibrils and favoring a decrease in shear force values.

#### 3.2.6. *capn1_4751*

The *capn1-4751* SNP (rs17872050) was located 24.17 kb from the start of the *capn1* gene. It exhibited strong LD (D′ > 0.95) with the *capn1_187* SNP discussed above (−1.86 kb) and some downstream SNPs, with the tender allele for *capn1_4751* corresponding to the tender alleles of these SNPs (results not shown). *capn1_189* SNP (+6.04 kb), *capn1_190* (+10.34 kb) and *capn1_191* (+13.79 kb) decreased the WBSF in late ageing by between 9.0% on d 14 and up to 16.2% on d 20, and selection for the *capn_4751* SNP would result in favorable responses in these tenderness phenotypes.

The SNP associated with the WBSF only between d 14 and d 20, resulting in a moderate tenderization of 0.47–0.50 kg shear force (Table 7), additive to the existing ageing tenderizing effects. The association between the *capn1-4751* SNP and MFL was, however, sustained from d 3 to d 14 post-mortem, although the effects on myofibril lengths were moderate. The MFL was decreased by 10.4% on d 3, 9.3% on d 9 and 8.1% on d 14 in *CC bulls compared to TT. These moderate gains were still important, as they occurred additive to the 32–33% decrease in the MFL than that which occurred between d 9 and d 20 of ageing, and the genotype effect deteriorated but did not disappear over time in late ageing as the myofibril lengths decreased. There was no advantage to adding breeds to the simplified mixed model as covariates.

The only other effect of the *capn1_4751* genotype on tenderizing phenotypes was an increase in the calpain-1 protease activity at 1 h post-mortem, although this effect was biologically small. The association with the protease was significant in the simplified model (*p* ≤ 0.0340), but only tended to be significant after adjustment for multiple testing (*p* ≤ 0.0769). The protease activity of *CC bulls was 7.7% greater than CT, with an intermediate increase in tender homozygotes compared to TT (Table 7).

## 4. Discussion

Of the total 134 SNPs tested in the gene-based association analyses, 62 SNPs showed some association with tenderness phenotypes in the WBSF or MFL in at least one of the ageing periods (*p* ≤ 0.10), while 38 SNPs associated significantly (*p* ≤ 0.05) with tenderness (results not shown). Only 12 of these SNPs, or less than 20%, associated with tenderness over extended periods of ageing, before quality control or biological importance of differences were considered. For these SNPs, it was more likely to identify associations from data collected at a single ageing period or from animals that showed variation in ageing, where 92% of the associations would be identified if any one ageing period was used to determine both the WBSF and MFL. However, more than 80% of the SNPs that could affect tenderness would likely fail to associate with tenderness from a single measurement, because they only associated with tenderness phenotypes for a short period of ageing. The least likely ageing period to identify an association in these data was d 9, where only 40% of all the possible SNPs (24/62) showed an association with the WBSF or MFL, while at the other ageing periods (d 3, d 14 and d 20), approximately 50–55% of all possible associations were identified.

Although the effects of genotypes in the calpain–calpastatin system are well-established, few studies have formally investigated the changes induced by ageing over time, especially when it came to using high-definition (777K) genotyping in South African beef cattle. Here, the SNPs in the *cast* and *capn2* gene that could sustain tenderizing effects for extended ageing periods were rare and did not have large effects on a variety of phenotypes of physical tenderness or protease activity. Previous studies similarly identified a limited number of 777K SNPs in the *capn1* and *cast* genes associated with tenderness, but these associations were limited to earlier ageing periods (≤d 7) and did not extend into the d 14 intermediate ageing period [68]. In our data, there were, however, a few SNPs in the *capn1* gene with larger sustained phenotypic responses in the tender genotypes (genotypes containing tender alleles) and could be linked physiologically to responses in the muscle calpain–calpastatin system.

Linkage disequilibrium (LD) is a requirement for effective genomic selection [69,70], but can also cause inadvertent negative effects from SNPs that are not targeted through selection [71], but linked in inheritance or pleiotropy [72]. Additionally, genotyping needs to be conducted at a sufficiently high density to identify LD between SNP markers [16,73,74] or the correlation in inheritance between alleles from different loci. The mean LD (D′) between the SNPs in the *cast* and *capn2* genes was high, 0.84 and 0.92, respectively, while greater levels of diversity (moderate D′) were present in the *capn1* (0.69) and the *capn3* (0.63) genes. All the SNPs in LD with the candidate SNPs for extended ageing were also linked to tender alleles that would result in responses that favored tenderness, with no apparent unintended negative effects on tenderization [71]. In addition to tenderness, other phenotypes such as the color, energy supply and water-holding capacity were recorded, but no clear adverse effects were identified through LD (results not shown).

It must be considered that the SNPs in this study were specifically chosen to sustain an effect on tenderness (WBSF or MFL) over time. If the three genotypes of an SNP responded to ageing in the same way, the association with the phenotype should remain relatively unaffected over the ageing period. One could assume that this should be true for the SNPs identified in this study, and this was generally true, with little difference in the absolute size of the tenderizing response (kg or µm) between d 3 and d 20, within each of the genotypes (bulls homozygous for the tender alleles, heterozygotes and bulls homozygous for the tough alleles).

If there was an interaction between the individual genotypes and tenderization over the ageing period, one genotype would tenderize more (or less) than the other genotypes over time. Longer ageing periods are generally employed to allow beef that tenderizes at a slower rate, to reach a greater tenderness before going to market. Over time, the differences between tough steaks in early ageing and those that tenderize rapidly by early ageing can decrease and even become negligible [9]. One could expect that it would become more difficult to identify genomic effects after longer periods of ageing (e.g., d 20), because the differences between the means of the toughest vs. most tender steaks decreased over time. An example of such an effect was shown in the *capn1_187* SNP, where the change in the MFL of the *TT and CT genotypes (containing the tender allele) was 11.93 µm and 13.53 µm, respectively (a 37–38% decrease in myofibril length to d 20). However, the change in the CC genotype was 20.3 µm or 45.8% between d 3 and d 20, and because the bulls with the “tough genotype” exhibited a greater response to ageing, the beneficial effect of the *T allele also gradually disappeared over time.

For this same SNP, however, (*capn1_187*), the effect on the WBSF was, generally, very stable and there was little difference between the responses of bulls with different genotypes and ageing for 20 days. Tender homozygous bulls (*TT) experienced a 2.22 kg reduction in the WBSF (38.2%), heterozygous bulls 2.32 kg (37.1%) and tough homozygous (CC) bulls 2.43 kg (35.7%). The differences between means of the genotypes, therefore, remained very stable over the different ageing periods, with little difference between d 3 and d 20 in the percentage difference of *TT compared to CC. This large tenderizing response, from very early ageing would be the ideal in a candidate SNP, because it would eliminate the financial and logistical costs associated with extended ageing; therefore, increasing profitability [11,12].

Conversely, the association of the *cast_736* SNP on the MFL changed dramatically over time from an effect of attenuated tenderization of *GG bulls on d 9, to a tenderizing effect of the same genotype on d 20. This dramatic change in the association between d 9 and d 20 was the result of an enhanced response to ageing in the *GG genotype, where the change in myofibril lengths of these bulls was 7.24 µm between d 9 and d 20, but only 4.1 µm in GT and 4.53 µm in TT.

The effects of the *cast*_*736* SNP on the WBSF that increased during ageing to a 10% decrease in shear force by d 14 in *GG bulls was promising. It was previously identified as a candidate SNP for selection for tenderness on approximately d 3 and d 14 of ageing [75]. The *cast_736* SNP fell within the range of a quantitative trait loci (QTL) for shear force identified previously, but was not one of the candidate SNPs tested by the 50K array used [76] and was also included in the GeneSeek HD Genomic Profiler (150K). Previous studies failed to associate the genotype of this SNP with tenderizing effects [68,77]. The moderate tenderizing response that was retained well into ageing could represent a meaningful gain in tenderness above either GT or TT bulls on d 20, if the proportion of *GG bulls could be increased through selection for this SNP. It would also have to be targeted specifically for selection, as it did not show LD with any other candidate SNPs in this experiment. To our knowledge, this is the first association of this SNP with tenderness phenotypes and confirmed a role for the SNP in the QTL that could be independent of the other SNPs in the QTL. However, the effects on the MFL and calpastatin that accompanied this improvement in the WBSF precluded the use of the SNP, as an unintended negative response in calpastatin inhibition (an increase) could result in a decreased proteolysis and increased toughening. The counterintuitive, toughening effect of the SNP on calpastatin in the “tender” genotype could explain the inconsistent effects in the MFL.

Although the present results showed small but persistent effects of the *cast_763* SNP on the WBSF, previous studies failed to identify an association between the SNP and tenderization [75]. Although the SNP fell within the location of a shear force QTL in the gene, the *cast_763* SNP was not included in the these analyses as a potential candidate SNP [76,78,79] or did not associate with tenderness [68,77]. Even though these effects were small, they proved statistically significant results in both models used to analyze these data. It follows that a small potential exists for an improvement in the marker through selection that would be linked to improvements in early and intermediate ageing through LD with neighboring SNPs, including measures of the WBSF, which were not significant for the SNP itself, as part of the QTL in this region of the gene [76]. This SNP was, however, already distributed toward the favorable allele, with a large proportion of *CC bulls (*n* = 113). This is characteristic of many SNPs in the *cast* gene [80,81,82], where the average tender allele frequency from all 77 SNPs genotyped in the *cast* gene averaged 71.3%, being the minor allele in only 9 (12%) of these SNPs. This could be the result of selection practices, as confirmed by the relatively high LD of the gene (D′ = 0.84).

The *capn2_780* SNP generally only tended to have a small effect on the MFL, but was persistent through most of the ageing periods tested. It was linked to SNPs that could favor responses in the muscle calpain system in this study that could indirectly enhance tenderization through proteolysis in selection. Although SNPs in the *capn2* gene were tested as possible candidates for selection for tenderness [83], this SNP has not yet been causally linked to tenderness [68,76,77].

The *capn1_184* SNP was located within QTL for tenderness phenotypes (shear force and panel score), where it was linked to the WBSF [68], while other studies found no association of the SNP with tenderness [75,76,78]. It was an excellent candidate for selection to improve tenderness over the entire ageing period for representative South African beef breeds, while positive gains would also be determined in the SNP through LD. Not only did the association of this SNP with the WBSF persist over the entire ageing period, but there were large gains during the intermediate ageing period (d 9 and d 14) and the 21–26% improvement in tenderness (≤d 14) was much greater than the 7% improvement in the WBSF or 9% in the MFL between d 14 and d20. This implied that beef could go to market sooner and this 1.1 kg difference in shear values between genotypes was large enough to make a considerable (perceptible) difference to the consumer. These results were in stark contrast with a previous study that failed to show an association of this SNP with direct or indirect measures of tenderness on d 3 or d 14 of ageing [75].

The *capn1_187* and *capn1_4751* SNPs were candidates for extended effects on tenderness throughout ageing in this study. They were slightly less effective than *capn1_184* in determining the WBSF, at least as effective for the MFL and had more pronounced effects on calpain protease activities. *capn1_4751* SNP was also linked to downstream SNPs that caused at least similar responses in tenderization. The *capn1-4751* SNP was the only one of the SNPs studied here that was identified as a causal SNP in QTL for shear force [76,79], and has been consistently included in research papers since its discovery in 2005 [84] and is included in most of the high-throughput arrays and commercial chips.

Although *capn1_187* was not identified as a candidate for selection for tenderness of beef [68,77], *capn1_4751* associated with several measures of tenderness in many studies. The *capn1-187* SNP was located within the range of QTL for shear force, but not included as a genotype in these research articles [76,78,79]. The large response in tenderization during early ageing of these SNPs was the ideal pattern of association, increasing tenderness rapidly within the first days of ageing, even though a diminishing effect of the genotype on the tenderness was noted in later ageing [47,48]. The association of the *capn1-187* SNP with tenderness phenotypes could arise from its strong LD with the causal SNP *capn1-4751*, through association in inheritance rather than an effect on the calpain-1 large subunit protein itself. The *capn1_4751* SNP has been shown to exhibit very low tender allelic frequencies in some Brahman [85], and both *capn1_4751* and *capn1_187* showed only 10% tender alleles in the Brahman breed, where the effect of the SNP remained tenderizing.

The present results agreed with that of previous studies [75], namely, that there were no significant interactions between breeds and genotype effects, although the phenotypes themselves were subject to breed effects. This meant that although there were differences between breeds for allelic frequencies, the associations of these SNPs with phenotypes remained intact within the different breeds and the SNPs could be used across different populations [75], as long as they remained polymorphic. This absence of breed effects could be the result of the conservation of these critical genes in evolution, because they perform a fundamental function as modulator proteins maintaining normal cellular function [86,87]. Because the six SNPs identified here could potentially increase tenderness in beef that was aged for as little as three days and up to three weeks, they could be effective for selection across different supply chains. Not only would the tenderness of rapidly tenderizing steaks be improved, but selection for these SNPs can improve the rate of tenderization of slow-ageing beef and across a variety of breed types. Although a larger dataset would be needed to confirm the results of these associations with tenderness, the increase in myofibrillar fragmentation and enhanced protease enzyme degradation associated with these SNPs made them good candidates for inclusion in custom selection arrays, in order to improve tenderization. It is, however, difficult to draw any firm conclusions from the relatively small number of animals genotyped here and detailed phenotypic data from a large number of animals subjected to high-density genotyping or sequencing [86,87] would be required to confirm these observations. Brahman-favorable allelic frequency was consistently below the average for all four genes, but particularly in *capn1,* where it was only 15.8% (vs. the average 50.8% tender alleles in *capn1*).

## 5. Conclusions

One possible way of decreasing the effective ageing period is through genomic selection for SNPs that (ideally) accelerate ageing over extended time periods. Because the SNPs of the calpain system are responsible for coding the most important proteases of tenderization and their inhibitor, these genes are likely targets for selection. However, the duration of ageing can alter genomic associations over time. Some of the SNPs in the muscle calpain system sustained genomic associations with tenderness throughout extended ageing periods, while some individual genotypes also responded differently to ageing, altering these associations over time. We found that ageing was accelerated in some tough genotypes in the *cast* and *capn1* genes, compared to genotypes that contained the tender alleles, progressively eroding genotype differences as the ageing was extended.

## Figures and Tables

**Table 1:** Descriptive statistics—means of the pooled data of all breeds and least squares means within breeds of production and tenderness data of South African feedlot-finished beef bulls.

	All Breeds (*n* = 166)		Angus (*n* = 24)	Bonsmara (*n* = 35)	Brahman (*n* = 35)	Charolais (*n* = 34)	Nguni (*n* = 35)	^#^ *p*-Value
BW (kg)	388 ± 4.26		422 ± 9.65 ^a^	399 ± 4.26 ^b^	393 ± 7.44 ^b^	423 ± 5.39 ^a^	308 ± 3.39 ^c^	0.0001
35dADG (kg/d)	1.54 ± 0.04		1.81 ± 0.08 ^b^	1.62 ± 0.05 ^ab^	1.37 ± 0.06 ^a^	1.88 ± 0.09 ^c^	1.21 ± 0.07 ^c^	0.0001
HCW (kg)	219 ± 2.46		234 ± 5.36 ^ab^	223 ± 2.87 ^b^	226 ± 4.67 ^b^	240 ± 3.44 ^a^	174 ± 2.07 ^c^	0.0001
CCW (kg)	215 ± 2.42		229 ± 5.29 ^ab^	218 ± 2.82 ^b^	221 ± 4.67 ^b^	234 ± 3.34 ^a^	170 ± 2.09 ^c^	0.0001
Dressing%	56.53 ± 0.15		55.62 ± 0.33 ^b^	55.96 ± 0.33 ^b^	57.44 ± 0.24 ^a^	56.64 ± 0.36 ^ab^	56.44 ± 0.34 ^ab^	0.0004
Mass Loss (%)	2.18 ± 0.03		2.16 ± 0.07	2.30 ± 0.06	2.22 ± 0.08	2.25 ± 0.07	2.14 ± 0.09	0.0564
EMA (mm^2^)	6 084 ± 64		5 853 ± 127 ^cd^	6 364 ± 134 ^ab^	5 922 ± 121 ^bc^	6 647 ± 127 ^a^	5 444 ± 120 ^d^	0.0001
WBSF (d 3)	6.15 ± 0.09		5.89 ± 0.20 ^b^	5.74 ± 0.20 ^b^	6.63 ± 0.19 ^a^	6.09 ± 0.21 ^ab^	6.17 ± 0.24 ^ab^	0.0122
WBSF (d 9)	4.83 ± 0.08	−21.4% ^1^	4.41 ± 0.16 ^b^	4.53 ± 0.15 ^b^	5.38 ± 0.21 ^a^	4.88 ± 0.19 ^ab^	4.75 ± 0.21 ^b^	0.0006
WBSF (d 14)	4.17 ± 0.07	−32.3% ^1^	3.75 ± 0.11 ^b^	4.20 ± 0.15 ^ab^	4.55 ± 0.15 ^a^	4.08 ± 0.16 ^ab^	3.99 ± 0.19 ^b^	0.0028
WBSF (d 20)	3.87 ± 0.07	−37.1% ^1^	3.64 ± 0.13 ^ab^	4.08 ± 0.15 ^a^	4.09 ± 0.15 ^a^	3.82 ± 0.14 ^ab^	3.52 ± 0.15 ^b^	0.0022
%ΔWBSF ^2^			−38.3%	−28.9%	−38.3%	−37.3%	−42.9%	
MFL (d 3)	35.51 ± 0.48		36.35 ± 1.17 ^ab^	31.74 ± 0.87 ^c^	39.65 ± 1.23 ^a^	35.53 ± 0.95 ^b^	32.93 ± 0.79 ^bc^	0.0001
MFL (d 9)	26.21 ± 0.31	−26.2% ^1^	25.55 ± 0.66 ^bc^	23.57 ± 0.47 ^c^	29.08 ± 0.84 ^a^	26.65 ± 0.73 ^b^	25.35 ± 0.49 ^c^	0.0001
MFL (d 14)	23.92 ± 0.28	−32.6% ^1^	22.87 ± 0.57 ^bc^	22.07 ± 0.44 ^c^	25.91 ± 0.71 ^a^	24.67 ± 0.79 ^ab^	23.72 ± 0.48 ^bc^	0.0001
MFL (d 20)	21.66 ± 0.23	−39.0% ^1^	20.74 ± 0.49 ^bc^	19.88 ± 0.40 ^c^	22.93 ± 0.54 ^a^	22.60 ± 0.57 ^a^	21.90 ± 0.45 ^ab^	0.0001
%ΔMFL ^2^			−42.9%	−37.4%	−42.2%	−36.4%	−33.5%	

Least squares means (LSM) ± standard errors (SE) that were significantly different between breeds (*p* ≤ 0.05) were indicated with different ascending letter superscripts (a, b, c) in each row. ^#^ *p*-values derived from a GLM; ^1^ the difference in tenderness at the ageing period, compared to d 3 post-mortem; ^2^ the difference in tenderness on d 20 compared to d 3 post-mortem within each breed; 35dADG—average daily gain recorded over the last 35 days before slaughter; BW—live body weight at slaughter; CCW—cold carcass weight; EMA—eye muscle area; HCW—hot carcass weight; MFL—myofibril fragment length (µm); WBSF—Warner–Bratzler shear force (kg).

**Table 2:** The effects of genotype of the *cast_736* SNP on the tenderness phenotypes, Warner–Bratzler shear force (WBSF) and myofibril fragment length (MFL) of representative purebred South African beef bulls during three to 20 days of ageing and on the muscle protease enzyme system during the first 20 h post-mortem.

*cast_736*	*GG (*n* = 19)	GT (*n* = 54)	TT (*n* = 93)	*p*-Value (Simplified)	Genotype Effect
WBSF (d 3)	5.90 ± 0.38 ^b^	6.05 ± 0.17 ^ab^	6.22 ± 0.12 ^a^	0.0130 ^$^	−5.1%
WBSF (d 9)	4.70 ± 0.33 ^b^	4.77 ± 0.14 ^ab^	4.92 ± 0.11 ^a^	0.0111 ^$^	−4.6%
WBSF (d 14)	3.78 ± 0.28 ^y^	4.17 ± 0.12 ^z^	4.21 ± 0.09 ^z^	0.0950 ^!^	−10.3%
WBSF (d 20)	3.63 ± 0.27	3.89 ± 0.12	3.90 ± 0.09	0.1343	−
MFL (d 3)	39.29 ± 1.89	34.51 ± 0.81	35.64 ± 0.61	0.4293	−
MFL (d 9)	28.20 ± 1.23 ^z^	25.36 ± 0.53 ^y^	26.50 ± 0.40 ^y^	0.0895	+6.4%
MFL (d 14)	24.42 ± 1.16 ^a^	23.17 ± 0.50 ^b^	24.20 ± 0.38 ^ab^	0.0074	^#^ −4.2%
MFL (d 20)	20.96 ± 0.94 ^b^	21.26 ± 0.41 ^b^	21.97 ± 0.31 ^a^	0.0448	−4.6%
calpastatin (1 h)	2.15 ± 0.10 ^z^	2.03 ± 0.04 ^y^	2.02 ± 0.03 ^y^	0.0811 ^!^	+6.4%
calpastatin (20 h)	1.87 ± 0.12	1.66 ± 0.05	1.72 ± 0.04	0.1819	−
calpain-1 (1 h)	1.53 ± 0.08	1.46 ± 0.04	1.40 ± 0.03	0.8459	−
calpain-1 (20 h)	1.17 ± 0.09	1.06 ± 0.04	1.10 ± 0.03	0.3287	−
calpain-2 (1 h)	1.02 ± 0.04	1.00 ± 0.02	0.98 ± 0.01	0.3944	−
calpain-2 (20 h)	1.03 ± 0.04	1.02 ± 0.02	1.01 ± 0.01	0.4279	−
calpastatin/calpain-1 (1 h)	1.40 ± 0.07 ^b^	1.44 ± 0.03 ^ab^	1.49 ± 0.02 ^a^	0.0398	−5.9%
calpastatin/calpain-1 (20 h)	1.66 ± 0.13	1.80 ± 0.06	1.68 ± 0.04	0.9142	−
calpastatin/calpains(1 h)	0.84 ± 0.03 ^a^	0.84 ± 0.01 ^a^	0.86 ± 0.01 ^a^	0.0105	n/a
calpastatin/calpains (20 h)	0.83 ± 0.04	0.82 ± 0.02	0.81 ± 0.01	0.1441	−

*GG—the genotype that favored tenderness; least squares means (LSM) ± standard errors (SE) that were significantly different within rows or ageing periods (*p* ≤ 0.05) were indicated with different ascending letter superscripts (a, b), and those that tended to be significantly different (*p* ≤ 0.10) with different descending letter superscripts (z, y). n/a—LSM differences were biologically negligible. ^#^ means difference of *GG compared to GT; ^$^ adjusted *p*-values only tended to be significant, ^!^ false positive association; MFL—myofibril fragment length; WBSF—Warner–Bratzler shear force.

**Table 3:** The effects of genotype of the *cast_763* SNP on the tenderness phenotypes, Warner–Bratzler shear force (WBSF) and myofibril fragment length (MFL) of representative purebred South African beef bulls during three to 20 days of ageing and on the muscle protease enzyme system during the first 20 h post-mortem.

*cast_763*	*CC (*n* = 113)	CT (*n* = 48)	^#^ TT (*n* = 5)	*p*-Value (Simplified)	Genotype Effect
WBSF (d 3)	5.99 ± 0.11	6.40 ± 0.17	−	0.5672	−
WBSF (d 9)	4.69 ± 0.09	4.97 ± 0.15	−	0.9146	−
WBSF (d 14)	4.09 ± 0.08	4.27 ± 0.13	−	0.7815	−
WBSF (d 20)	3.81 ± 0.08	3.93 ± 0.12	−	0.9412	−
MFL (d 3)	34.77 ± 0.52 ^y^	36.56 ± 0.85 ^z^	−	0.0902	−4.9%
MFL (d 9)	25.64 ± 0.35 ^b^	27.14 ± 0.57 ^a^	−	0.0289	−5.5%
MFL (d 14)	23.38 ± 0.33 ^b^	24.45 ± 0.54 ^a^	−	0.0005	−4.4%
MFL (d 20)	21.28 ± 0.27 ^b^	22.30 ± 0.43 ^a^	−	0.0081	−4.6%
calpastatin (1 h)	1.96 ± 0.03 ^y^	2.07 ± 0.04 ^z^	−	0.0828 ^!^	−5.5%
calpastatin (20 h)	1.63 ± 0.03	1.76 ± 0.05	−	0.1042	−
calpain-1 (1 h)	1.40 ± 0.02 ^z^	1.42 ± 0.04 ^z^	−	0.0848 ^!^	n/a
calpain-1 (20 h)	1.04 ± 0.03	1.09 ± 0.04	−	0.3695	−
calpain-2 (1 h)	0.98 ± 0.01	1.00 ± 0.02	−	0.4187	−
calpain-2 (20 h)	1.01 ± 0.01	1.03 ± 0.02	−	0.5901	−
calpastatin/calpain-1 (1 h)	1.44 ± 0.02	1.50 ± 0.03	−	0.9886	−
calpastatin/calpain-1 (20 h)	1.69 ± 0.04	1.77 ± 0.06	−	0.4553	−
calpastatin/calpains(1 h)	0.83 ± 0.01	0.87 ± 0.02	−	0.5752	−
calpastatin/calpains (20 h)	0.80 ± 0.01 ^y^	0.85 ± 0.02 ^z^	−	0.0876	−5.6%

*CC—the genotype that favored tenderness; least squares means (LSM) ± standard errors (SE) that were significantly different within rows or ageing periods (*p* ≤ 0.05) were indicated with different ascending letter superscripts (a, b), and those that tended to be significantly different (*p* ≤ 0.10) with different descending letter superscripts (z, y). ^#^ Tough homozygotes were not available for comparison; ^!^ adjusted *p*-values identified a false positive association; MFL—myofibril fragment length; WBSF—Warner–Bratzler shear force.

**Table 4:** The effects of genotype of the *capn2_780* SNP on the tenderness phenotype, Warner–Bratzler shear force (WBSF) of representative purebred South African beef bulls during three to 20 days of ageing and on the muscle protease enzyme system during the first 20 h post-mortem.

*capn2_780*	*AA (*n* = 112)	AG (*n* = 39)	^#^ GG (*n* = 15)	*p*-Value (Simplified)	Genotype Effect
WBSF (d 3)	6.05 ± 0.11	6.54 ± 0.20	−	0.3279	−
WBSF (d 9)	4.77 ± 0.09	4.83 ± 0.17	−	0.1467	−
WBSF (d 14)	4.10 ± 0.08	4.30 ± 0.15	−	0.2274	−
WBSF (d 20)	3.84 ± 0.08	3.93 ± 0.14	−	0.3831	−
calpastatin (1 h)	2.06 ± 0.03	1.87 ± 0.05	−	0.9969	−
calpastatin (20 h)	1.75 ± 0.03	1.48 ± 0.06	−	0.7869	−
calpain-1 (1 h)	1.45 ± 0.02	1.35 ± 0.04	−	0.3737	−
calpain-1 (20 h)	1.10 ± 0.03	0.99 ± 0.05	−	0.3225	−
calpain-2 (1 h)	1.00 ± 0.01	0.95 ± 0.02	−	0.9139	−
calpain-2 (20 h)	1.03 ± 0.01	0.96 ± 0.02	−	0.6730	−
calpastatin/calpain-1 (1 h)	1.47 ± 0.02	1.42 ± 0.04	−	0.7586	−
calpastatin/calpain-1 (20 h)	1.74 ± 0.04	1.62 ± 0.07	−	0.9579	−
calpastatin/calpains(1 h)	0.85 ± 0.01	0.82 ± 0.02	−	0.7954	−
calpastatin/calpains (20 h)	0.83 ± 0.01	0.77 ± 0.02	−	0.7862	−

*AA—the genotype that favored tenderness; least squares means (LSM) ± standard errors (SE) were not significantly different (*p* > 0.10). ^#^ Tough homozygotes were not available for comparison; WBSF—Warner–Bratzler shear force.

**Table 5:** The effects of genotype of the *capn1_184* SNP on the tenderness phenotype, myofibril fragment length (MFL) of representative purebred South African beef bulls during three to 20 days of ageing and on the muscle protease enzyme system during the first 20 h post-mortem.

*capn1_184*	*GG (*n* = 77)	AG (*n* = 66)	AA (*n* = 23)	*p*-Value (Simplified)	Genotype Effect
MFL (d 3)	33.47 ± 0.65	35.48 ± 0.72	41.57 ± 1.74	0.1810	−
MFL (d 9)	24.67 ± 0.42 ^c^	26.20 ± 0.47 ^b^	31.98 ± 1.12 ^a^	0.0242	−22.9%
MFL (d 14)	22.50 ± 0.38 ^y^	24.05 ± 0.42 ^y^	30.54 ± 1.02 ^z^	0.0771	−26.3%
MFL (d 20)	20.92 ± 0.31	21.54 ± 0.35	25.70 ± 0.84	0.1557	−
calpastatin (1 h)	1.99 ± 0.04	1.99 ± 0.04	2.23 ± 0.09	0.4096	−
calpastatin (20 h)	1.69 ± 0.04	1.64 ± 0.05	1.81 ± 0.11	0.6957	−
calpain-1 (1 h)	1.47 ± 0.03	1.41 ± 0.03	1.32 ± 0.08	0.2095	−
calpain-1 (20 h)	1.13 ± 0.03	1.05 ± 0.04	0.97 ± 0.09	0.7597	−
calpain-2 (1 h)	1.00 ± 0.01	1.00 ± 0.01	0.98 ± 0.03	0.7125	−
calpain-2 (20 h)	1.04 ± 0.01	1.02 ± 0.01	0.98 ± 0.04	0.5729	−

*GG—the genotype that favored tenderness; least squares means (LSM) ± standard errors (SE) that were significantly different within rows or ageing periods (*p* ≤ 0.05) were indicated with different ascending letter superscripts (a, b, c), and those that tended to be significantly different (*p* ≤ 0.10) with different descending letter superscripts (z, y). MFL—myofibril fragment length.

**Table 6:** The effects of genotype of the *capn1_187* SNP on the tenderness phenotype, myofibril fragment length (MFL) of representative purebred South African beef bulls during three to 20 days of ageing and on the muscle protease enzyme system during the first 20 h post-mortem.

*capn1_187*	*TT (*n* = 76)	CT (*n* = 57)	CC (*n* = 33)	*p*-Value (Simplified)	Genotype Effect
MFL (d 3)	32.54 ± 0.64 ^c^	35.33 ± 0.76 ^b^	44.32 ± 2.10 ^a^	0.0090	−26.6%
MFL (d 9)	24.30 ± 0.43 ^c^	26.23 ± 0.51 ^b^	29.71 ± 1.40 ^a^	0.0047	−18.2%
MFL (d 14)	22.25 ± 0.40	24.38 ± 0.47	26.65 ± 1.30	0.1270	−
MFL (d 20)	20.61 ± 0.32	21.80 ± 0.38	24.02 ± 1.06	0.1885	−
calpastatin (1 h)	2.00 ± 0.04	2.04 ± 0.04	1.96 ± 0.12	0.2181	
calpastatin (20 h)	1.73 ± 0.04 ^z^	1.69 ± 0.05 ^z^	1.61 ± 0.14 ^z^	0.0640 ^!^	^#^ variable
calpastatin/calpain-1 (1 h)	1.38 ± 0.02 ^z^	1.44 ± 0.03 ^z^	1.63 ± 0.08 ^y^	0.0909	−15.5%
calpastatin/calpain-1 (20 h)	1.63 ± 0.05	1.69 ± 0.06	1.84 ± 0.16	0.1571	
calpastatin/calpains (1 h)	0.81 ± 0.01	0.83 ± 0.01	0.90 ± 0.04	0.1989	
calpastatin/calpains (20 h)	0.79 ± 0.01	0.80 ± 0.02	0.85 ± 0.04	0.5493	

*TT—the genotype that favored tenderness; least squares means (LSM) ± standard errors (SE) that were significantly different within rows or ageing periods (*p* ≤ 0.05) were indicated with different ascending letter superscripts (a, b, c), and those that tended to be significantly different (*p* ≤ 0.10) with different descending letter superscripts (z, y). ^#^ Responses of the *TT genotype were variable between breeds and not always tenderizing, ^!^ false positive association; MFL—myofibril fragment length.

**Table 7:** The effects of genotype of the *capn1_4751* SNP on the tenderness phenotypes, Warner–Bratzler shear force (WBSF) and myofibril fragment length (MFL) of representative purebred South African beef bulls during 3 to 20 days of ageing and on the muscle protease enzyme system during the first 20 h post-mortem.

*capn1_4751*	*CC (*n* = 53)	CT (*n* = 63)	TT (*n* = 50)	*p*-Value (Simplified)	Genotype Effect
WBSF (d 3)	5.89 ± 0.16	6.17 ± 0.15	6.26 ± 0.22	0.3758	−
WBSF (d 9)	4.47 ± 0.14	4.94 ± 0.13	4.85 ± 0.19	0.3035	−
WBSF (d 14)	3.84 ± 0.12 ^b^	4.14 ± 0.11 ^ab^	4.31 ± 0.17 ^a^	0.0357	−10.8%
WBSF (d 20)	3.58 ± 0.11 ^b^	3.88 ± 0.11 ^ab^	4.07 ± 0.16 ^a^	0.0226	−12.0%
MFL (d 3)	32.43 ± 0.79 ^y^	35.72 ± 0.73 ^z^	36.17 ± 1.09 ^z^	0.0790	−10.4%
MFL (d 9)	24.20 ± 0.52 ^b^	26.14 ± 0.48 ^a^	26.69 ± 0.72 ^a^	0.0474	−9.3%
MFL (d 14)	22.11 ± 0.49 ^y^	24.36 ± 0.45 ^z^	24.07 ± 0.67 ^z^	0.0762	−8.1%
MFL (d 20)	20.32 ± 0.40	21.90 ± 0.37	21.94 ± 0.55	0.1056	−
calpastatin (1 h)	2.02 ± 0.04	2.03 ± 0.04	2.08 ± 0.06	0.1216	−
calpastatin (20 h)	1.73 ± 0.05	1.73 ± 0.05	1.74 ± 0.07	0.1424	−
calpain-1 (1 h)	1.54 ± 0.03 ^a^	1.43 ± 0.03 ^b^	1.48 ± 0.05 ^ab^	0.0340 ^$^	+4.1%
calpain-1 (20 h)	1.14 ± 0.04	1.13 ± 0.04	1.08 ± 0.05	0.1847	−
calpain-2 (1 h)	1.03 ± 0.01	1.00 ± 0.01	1.03 ± 0.02	0.2252	−
calpain-2 (20 h)	1.05 ± 0.02	1.04 ± 0.01	1.03 ± 0.02	0.3517	−
calpastatin/calpain-1 (1 h)	1.35 ± 0.03	1.45 ± 0.03	1.46 ± 0.04	0.3813	−
calpastatin/calpain-1 (20 h)	1.64 ± 0.06	1.69 ± 0.05	1.77 ± 0.08	0.7056	−
calpastatin/calpains (1 h)	0.79 ± 0.01	0.84 ± 0.01	0.84 ± 0.02	0.8265	−
calpastatin/calpains (20 h)	0.79 ± 0.02	0.80 ± 0.02	0.83 ± 0.02	0.3613	−

*CC—the genotype that favored tenderness; least squares means (LSM) ± standard errors (SE) that were significantly different within rows or ageing periods (*p* ≤ 0.05) were indicated with different ascending letter superscripts (a, b), and those that tended to be significantly different (*p* ≤ 0.10) with different descending letter superscripts (z, y). ^$^ adjusted *p*-values only tended to be significant; MFL—myofibril fragment length; WBSF—Warner–Bratzler shear force.

## Data Availability

The data presented in this study are available on request from the corresponding author.
